# Therapist's Assessment of Their Patient's Session‐Level Emotional Processes: Validation of the In‐Session Patient Affective Reactions Questionnaire–Clinician Form

**DOI:** 10.1002/jclp.70023

**Published:** 2025-08-06

**Authors:** Alberto Stefana, Buket Ünver, Eduard Vieta, Paolo Fusar‐Poli, Eric A. Youngstrom

**Affiliations:** ^1^ Department of Brain and Behavioral Sciences University of Pavia Pavia Italy; ^2^ Center for Behavioural Sciences and Mental Health National Institute of Health Rome Italy; ^3^ Department of Psychology, Faculty of Economics, Administrative and Social Sciences Işık University Istanbul Turkey; ^4^ Bipolar and Depressive Disorders Unit, Hospital Clinic, IDIBAPS, CIBERSAM University of Barcelona Barcelona Spain; ^5^ OASIS Service South London and Maudsley NHS Foundation Trust London UK; ^6^ Early Psychosis: Interventions and Clinical‐detection (EPIC) Lab, Department of Psychosis Studies, Institute of Psychiatry, Psychology & Neuroscience King's College London London UK; ^7^ Institute for Mental and Behavioral Health Research, Nationwide Children's Hospital, Division of Child and Family Psychiatry The Ohio State University Columbus Ohio USA; ^8^ Department of Psychology and Neuroscience University of North Carolina at Chapel Hill Chapel Hill North Carolina USA; ^9^ Helping Give Away Psychological Science Chapel Hill North Carolina USA

**Keywords:** affective reaction, assessment, emotional reaction, in‐session process, psychotherapy, therapeutic relationship, therapist‐report measure

## Abstract

**Background:**

The current study aimed to evaluate a therapist version of the *in‐Session Patient Affective Reactions Questionnaire* (SPARQ). The SPARQ was developed to assess a pattern of emotions, thoughts, and behaviors experienced by a patient toward their therapist during a session. The SPARQ has existed only as a patient self‐report measure and has demonstrated promise as a psychotherapy process measure. This study intended to validate a complementary clinician‐report version of the questionnaire: the SPARQ‐C.

**Methods:**

A sample of licensed mental health clinicians (*N* = 151) completed the SPARQ‐C along with other measures. Data analysis involved exploratory and confirmatory factor analyses (CFA). Reliability and convergent and criterion‐related validity of the SPARQ‐C were also evaluated.

**Results:**

The SPARQ‐C preserved the two‐factor structure: positive affect (*k* = 4, *ω* total = 0.84) and negative affect (*k* = 4, *ω* total = 0.70), which correlated *r* = 0.26. CFA using the a priori model two‐factor model based on the patient‐report version provided the following fit indices: χ^2^_[19]_ = 26.01, CFI = 0.98; TLI = 0.97, RMSEA = 0.05 (90% CI [0.00, 0.09]), and SRMR = 0.05. The SPARQ‐C scales demonstrated convergent and criterion‐related validity with measures of other elements of the therapeutic relationship, session outcome, and demographic‐clinical variables.

**Discussion:**

The SPARQ‐C is a reliable measure suitable for both clinical and research purposes. It allows for a nuanced assessment of patients' session‐level affective responses towards their therapist from the clinician's perspective.

## 1 Introduction

Emotions constitute an essential aspect of human existence, stemming from an evolutionary process designed to enable individuals to respond effectively to significant interpersonal interactions and critical life challenges (Ekman 2005; Keltner et al. 2019). Given their pivotal role, it is understandable that emotions have been recognized as therapeutically important since the inception of psychotherapy (Irarrázaval and Kalawski 2022; Stefana 2015). This recognition extends to both the emotional expressions of patients and the emotional experiences of therapists as they endeavor to provide therapeutic assistance (Abargil and Tishby 2021; Diaz et al. 2023).

Research on the role of emotions in psychotherapy has demonstrated that patients' emotions significantly contribute to both session and treatment outcomes (Peluso and Freund 2018; Sønderland et al. 2023). However, it is not just the patients' emotions that are important; the clinicians' emotions are also pivotal. Evidence indicates that therapists' openness to experiencing different emotional states during sessions (Abargil and Tishby 2021) and their ability to manage emotional reactions based on their unresolved personal conflicts, triggered by interactions with the patient (Hayes et al. 2018) (Hayes et al. 2018), are important elements in achieving positive therapy outcomes.

Emotions, which along with cognitions form integrated wholes (Colombetti 2007; Izard et al. 2006), are a key element of psychotherapy processes related to its effectiveness and efficacy (Faustino 2022; Lane et al. 2022). Facilitating patients' awareness of their emotional states, improving their expression, and making constructive use of them are key to therapeutic change (Greenberg 2008, 2012). To achieve this, the clinician's empathic and aware understanding of the patient is essential, as feeling emotionally understood promotes patients' emotional well‐being (Atzil‐Slonim et al. 2019) and can facilitate them to understand their emotional states (Irarrázaval and Kalawski 2022). Therefore, clinicians should continuously and collaboratively bring into sharp focus the emotions experienced by the patient in the here and now of the session, helping them to feel, explore, recognize, accept, elaborate, transform, and manage these emotions (Greenberg and Goldman 2019).

Counseling and psychotherapy are interpersonal experiences that can reactivate denied memories and associated repressed emotions, as well as elicit new emotional reactions. Both events provide valuable insights into patients' needs and patterns of reaction to specific interpersonal and intrapersonal situations, offering a chance to address emotions that were previously feared and denied and to integrate new, positive experiences with negative ones from the past (Greenberg 2019). This helps patients transform persistent, memory‐based problematic emotional patterns and, in turn, change their maladaptive patterns of interpersonal interactions (Lane et al. 2015). Among the various emotions experienced by patients during therapy sessions, those directed towards the clinician are particularly significant and instrumental in facilitating the therapeutic work described above (Høglend 2014; Subic‐Wrana et al. 2016). An empathic and supportive relationship with the therapist is crucial in determining whether emotional experiences and expressions are constructive or detrimental to the patient (Peluso and Freund 2018). Such a relationship is both a fundamental component of therapeutic change and a prerequisite for the implementation of effective psychotherapy interventions (Hayes and Vinca 2017).

Therefore, it is crucial for therapists to regularly monitor even subtle changes in a patient's emotional perception of the therapeutic relationship to address and mend any disruptions. Without monitoring the fluctuations in a patient's emotions, timely and appropriate interventions cannot be made. In this light, we developed the in‐Session Patient Affective Reactions Questionnaire (SPARQ) (Stefana et al. 2023).

### 1.1 An Overview of the SPARQ Development

The development and validation of the SPARQ followed best practice recommendations (DeVellis and Thorpe 2022; Stefana et al. 2025a) and are detailed elsewhere (Stefana et al. 2023, 2024a). The phenomenon we aimed to measure was the pattern of emotional, cognitive, and behavioral reactions that a patient experiences toward their therapist during a counseling or psychotherapy session—reactions that stem from the patient‐therapist interaction. This construct includes transference (i.e., reactions based on the patient's unresolved conflicts) but is much more comprehensive, implying that not all reactions are transferential.

The design of the SPARQ was guided by several theoretical frameworks, notably the hierarchical model of affect (Tellegen et al. 1999a, 1999b) and the social and interpersonal dimension of the dominance behavioral system (Johnson et al. 2012). In addition, the tripartite model of the psychotherapy relationship (Gelso 2014) and the contextual model (Wampold 2015) were incorporated to ensure the instrument's relevance and practical application in clinical settings. Both inductive and deductive methods were employed to develop the initial item pool. We reviewed established scales (e.g., Bradley et al. 2005; Izard et al. 1991, 1993; Kelley et al. 2010; Multon et al. 1996) and selected or adapted items as needed to capture the full range of reactions that adult patients may experience during therapy sessions. Items were crafted to assess both positive and negative affective domains while ensuring unidimensionality.

The initial pool consisted of 131 items, which were administered to 701 adult psychotherapy patients with a psychiatric diagnosis (Stefana et al. 2023). We used parallel analysis to determine the number of factors to extract, which indicated a six‐factor structure. Exploratory factor analysis (EFA) was conducted accordingly, with factors assessed for clarity and consistency. Items that loaded onto multiple factors without a clear fit were removed. After several rounds of EFA, we refined the measure to 68 items, revealing a three‐factor structure. Items within the first factor had lower response rates, creating a skewed distribution. Based on clinical relevance, we recoded these items into a simpler, two‐option format and analyzed them separately, forming the Rift In‐Session Questionnaire (RISQ). For the remaining two factors, we applied an item response theory model to select items that covered a full range of responses. The final SPARQ measure includes two four‐item scales: one for positive affect and one for negative affect.

The 8‐item SPARQ was then administered to a different sample of 700 adult psychotherapy patients, with or without a psychiatric diagnosis (Stefana et al. 2024a). Confirmatory factor analysis demonstrated excellent fit indices, and internal reliability was confirmed. The positive affect scale demonstrated strong correlations with the working alliance construct—especially its bond dimension—and with the addressing of alliance ruptures during the session. It also showed a moderate correlation with the real relationship construct and was strongly correlated with session outcome measures. The negative affect scale was moderately and negatively correlated with alliance quality, the real relationship, and session outcomes. It was also moderately correlated with the magnitude of tension experienced by the patient toward the therapist during the session.

In an initial attempt to understand the interconnections among the constructs of positive and negative affects as measured by the SPARQ and those of real relationship and working alliance, we conducted a principal‐axis factor analysis that revealed four factors closely corresponding to those posited for the respective scales (Stefana et al. 2024b). There were some interesting departures from expectation, too. Notably, items of the bond dimension of the working alliance loaded on the same factor as items from the SPARQ positive affect scale but did not align with the factor comprising real relationship items. Goal and task items of the working alliance loaded on their own factor. These findings suggest that the SPARQ measures key elements of the therapeutic relationship while offering complementary information beyond existing measures of real relationship and cognitive components of the working alliance, specifically by capturing session‐level affective reactions of the patient toward their therapist.

### 1.2 The Current Study

The absence of a therapist version of the SPARQ might limit existing research efforts, providing data on session‐level affective responses of the patients towards their therapist only from the perspective of the patient. Given this, a clinician version of the SPARQ can enhance understanding of the affective processes in dyadic therapy relationships, as well as add to the nomothetic network for the construct of transference (Racker 2018; Westen and Gabbard 2002), elucidating its relationship with session and treatment outcomes. The present study aimed to evaluate the psychometric properties and validity of a therapist version of the SPARQ. It was hypothesized that this version would mirror the patient version in terms of factor structure and content. Based on previous findings with the patient version (Stefana et al. 2023), we expected moderate correlations between the SPARQ clinician form and measures of alliance, real relationship, and session outcome. Additionally, higher correlation coefficients were anticipated for the positive affect scale compared to the negative affect scale (again, following patterns observed with patient‐report; Stefana et al. 2023).

## 2 Methods

### 2.1 Participants

A total of 151 mental health clinicians participated in the study, with a majority being female (71%, *n* = 108). The most represented age group was 60 years and older (57%, *n* = 86), followed by those aged 50 to 59 years (23%, *n* = 35). In terms of psychotherapeutic orientations, the largest group practiced psychodynamic/psychoanalytic therapy (40%, *n* = 61), while behavioral therapy was represented by 19% (*n* = 28) of the sample. More than half of the clinicians had over 20 years of post‐licensure clinical experience (55%, *n* = 83) and dedicated more than 20 h per week to therapy (46%, *n* = 70). Each clinician provided data for one patient. Among the patients, 65% (*n* = 98) were female and 47% (*n* = 71) were aged between 30 and 49 years. Most patients (75%, *n* = 113) had a psychiatric disorder diagnosis, with the Clinical Global Impression scale (Busner and Targum 2007) rating them as mildly (33%, *n* = 50) or moderately (25%, *n* = 37) ill. The mean Global Assessment of Functioning (Jones et al. 1995) score was 67.4 (SD = 12.4). Approximately half of the patients had been receiving psychotherapy for over 2 years (48%, *n* = 73), typically attending one session per month (48%, *n* = 72). Table 1 details the demographics and professional characteristics of the clinicians, while Table 2 provides the demographics and clinical data of the patients, as well as treatment characteristics.

**Table 1:** Therapist demographic and professional characteristics (*N* = 151).

	**% (*n*)**
**Demographic characteristics**	
Age (years)	
30–39	5% (8)
40–49	15% (22)
50–59	23% (35)
60 or above	57% (57)
Gender	
Woman	72% (108)
Man	28% (43)
Ethnicity	
White	91% (138)
Other	9% (13)
**Professional Characteristics**	
Professional background	
Psychologist	36% (54)
Psychiatrist	13% (20)
Social Worker	21% (31)
Other	31% (46)
Psychotherapeutic approach	
Behavior therapy	19% (28)
Cognitive therapy	7% (11)
Humanistic therapy	7% (11)
Integrative or holistic therapy	9% (13)
Psychoanalysis or psychoanalytic therapy	27% (41)
Psychodynamic therapy	13% (20)
Other	18% (27)
Post‐licensed experience (years)	
1 to 6	6% (10)
7 to 10	9% (14)
11 to 15	15% (22)
16 to 20	15% (22)
More than 20	55% (83)
Time spent practicing therapy (hours per week)	
1 to 5	7% (10)
6 to 10	11% (17)
11 to 15	13% (19)
16 to 20	23% (35)
21 or more	46% (70)

**Table 2:** Patient demographic, clinical, and treatment characteristics (*N* = 151).

	**% (*n*)**
**Demographic characteristics**	
Age (years)	
18–22	6% (9)
23–29	17% (25)
30–39	22% (34)
40–49	24% (37)
50–59	17% (25)
60 or above	14% (21)
Gender	
Woman	64% (98)
Man	32% (48)
Nonbinary/third gender	3% (5)
Ethnicity	
White	82% (123)
Asian	5% (8)
Other	13% (20)
**Clinical Characteristics**	
Presence of any psychiatric mental disorder	75% (113)
Any anxiety disorder	42% (64)
Any trauma‐ and stressor‐related disorders	21% (31)
Any (unipolar) depressive disorder	20% (30)
Any neurodevelopmental disorder	8% (12)
Any personality disorder	6% (9)
Any eating disorder	5% (8)
Any bipolar or related disorder	5% (7)
Any other disorder	6% (9)
CGI	
Normal, not at all ill	21% (32)
Borderline mentally ill	19% (28)
Mildly ill	33% (50)
Moderately ill	25% (37)
Markedly/Severely ill	2% (4)
GAF, *M (SD)*	67.4 (12.4)
**Treatment Characteristics**	
Therapy length (months)	
0 to 3	13% (20)
4 to 6	8% (12)
7 to 12	13% (19)
13 to 24	18% (27)
24 or more	48% (73)
Session frequency	
≤ 1 per month	10% (15)
2 to 3 per month	30% (46)
1 per week	48% (72)
≥ 2 per week	12% (18)
Session attendance	
In person	52% (78)
Telephone call	2% (4)
Video call	46% (69)
Location	
Private practice	89% (134)
Private health institution	7% (11)
Other	4% (6)

### 2.2 Measures

A broad spectrum of self‐report instruments collected detailed data on clinicians' and patients' individual characteristics, the therapeutic relationship dynamics, and session outcomes.

### 2.3 Demographic and Therapy Domain

#### 2.3.1 Clinician Demographic and Professional Data Form

Clinicians completed a demographic and professional data form, capturing the information shown in Table 1.

#### 2.3.2 Patient Demographic and Clinical Data Form

Clinicians reviewed their appointment records to identify the last adult patient (18 years or older) seen for an individual session. They completed a demographic and clinical data form for that patient, which included data reported in Table 2. The form encompassed assessments using two validated measures of symptom severity: the Global Assessment of Functioning (GAF) scale (Jones et al. 1995) and the Clinical Global Impressions (CGI) scale (Busner and Targum 2007).

### 2.4 Therapeutic Relationship Domain

#### 2.4.1 In‐Session Patient Affective Reactions Questionnaire–Clinician (SPARQ‐C) Form

Originally developed as a self‐report measure for patient perceptions and affective reactions (Stefana et al. 2023), the SPARQ has been validated for clinician ratings in this study. Each item was rephrased from the clinician's perspective. This involved changing the subject of the sentence from the patient to the clinician. For example, the item “I felt happy to see my therapist” was modified to “They felt happy to see me.”

The SPARQ‐C (Stefana et al. 2023, 2024a) consists of two scales, each with four items: positive affect and negative affect. The positive affect scale evaluates the patient's feelings of security and comfort within the therapeutic relationship. The negative affect scale evaluates the patient's feelings of shame, shyness, fear of speaking openly, worry about receiving inadequate help from the therapist, and a sense of failure. Each item is rated on a 5‐point Likert scale from 0 (‘Not at all true’) to 4 (‘Very true’). The patient form of the SPARQ positive affect scale demonstrated convergent validity with the Real Relationship–Client form (*r* = 0.52), and the goal (*r* = 0.68), task (*r* = 0.67), and bond subscale (*r* = 0.83) of the patient version of the WAI‐SR. The negative affect scale showed a pattern of correlations similar to the positive affect scale but in the opposite direction and with reduced intensity (*r* coefficients ranging from −0.40 to −0.46) (Stefana et al. 2024a). In this study, the SPARQ‐C showed McDonald's *ω* total coefficients of 0.83 for positive affect and 0.78 for negative affect.

#### 2.4.2 Therapy Session Checklist–Transference Items (TSC‐TI)

The TSC‐TI consists of three single items from the Therapist Session Checklist (Graff and Luborsky 1977). Clinicians are asked to rate the patient's transference—defined as the displacement of material from an early significant relationship onto the clinician—using a five‐point scale from 1 (‘None or slight’) to 5 (‘Very much’). The construct validity of the TSC‐TI is supported by the association between its scores and phenomena such as counselor intentions, successful analysis, and a multi‐item transference measure (Gelso et al. 1991; Graff and Luborsky 1977; Multon et al. 1996). Internal consistency does not apply to single‐item scales.

#### 2.4.3 Working Alliance Inventory–Short Revised–Therapist Version (WAI‐SR‐T)

The WAI‐SR‐T (Hatcher et al. 2020) is an instrument that assesses the quality of the working alliance from the perspective of the clinician. It comprises three subscales, four items each, assessing patient‐clinician agreement on therapy tasks, therapy goals, and the strength of the affective bond. Each item is rated on a 7‐point Likert scale from 0 (‘Not at all’) to 5 (‘Completely’). Higher scores indicate a better quality of alliance. In this study, the internal consistency Cronbach's *α* for the total scale was 0.88, while the goals, tasks, and bond subscales showed McDonald's ω total values of 0.79, 0.84, and 0.80, respectively.

#### 2.4.4 Real Relationship Inventory–Therapist (RRI‐T) Form

The RRI‐T (Gelso et al. 2005) is a 24‐item scale that measures the strength of the real relationship between patient and therapist, with the latter rating themselves, the patient, and their relationship on a five‐point Likert scale ranging from 1 (‘Strongly disagree’) to 5 (‘Strongly agree’). It comprises two subscales, 12 items each, named realism and genuineness. Realism measures the degree of realistic perception of the other, while genuineness assesses authenticity and the extent of being genuinely oneself. Higher scores signify stronger real relationships. Construct validity was demonstrated through the associations with multiple elements of the therapeutic relationship, including working alliance, and measures of session/treatment outcomes (Bhatia and Gelso 2018; Stefana et al. 2024b). In this study, the internal consistency McDonald's *ω* total values were 0.83 for realism and 0.84 for genuineness, with Cronbach's α of 0.84 for the total scale.

#### 2.4.5 Clinician Affective Response (CARE) Scale

The CARE scale (Stefana et al. 2024c, 2025b) is a 15‐item self‐report inventory that evaluates clinicians' emotional, cognitive, and behavioral responses toward their patients during individual adult psychotherapy sessions. Responses are rated on a 3‐point Likert scale: 0 = “Not at all,” 1 = “A little,” and 2 = “Somewhat‐to‐Very much.” The CARE scale comprises three five‐item subscales: positive engagement, enmeshed, and stuck. The positive engagement subscale reflects feelings of appreciation for the patient, contentment, comfort, enthusiasm, and interest in their work. The enmeshed subscale measures a desire to give love, act for the patient, concern for their feelings and needs, tenderness, and a tendency to protect them. The stuck subscale describes difficulty entering the patient's inner world, frustration, feelings of incompetence, hopelessness, and annoyance. Because it measures three distinct constructs with low correlations, the CARE scale does not provide a total score. In this study, the scale's internal consistency was McDonald's *ω* total of 0.78 for positive engagement, 0.72 for enmeshed, and 0.71 for stuck.

#### 2.4.6 Inventory of Countertransference Behavior (ICB)

The ICB, originally an observer‐rated measure (Friedman and Gelso 2000), has been validated as a self‐report measure of countertransference behavior (Bhatia and Gelso 2018). Its 21 items are rated on a five‐point scale from 1 (‘To little or no extent’) to 5 (‘To a great extent’), with higher scores indicating more countertransference. The ICB provides three scores: negative, positive, and overall countertransference. It has demonstrated reliability and validity through meaningful associations with constructs like countertransference management and working alliance, as well as associations with measures of therapy outcomes (Bhatia and Gelso 2018; Mohr et al. 2005). In this study, internal consistency was McDonald's *ω* total of 0.66 for positive countertransference, 0.68 for negative countertransference, and Cronbach's *α* of 0.81 for the total measure.

### 2.5 Session Outcome Domain

#### 2.5.1 Session Quality

This clinician‐rated single‐item scale evaluates the overall session quality (Gelso et al. 1991). The item is rated on a five‐point scale from 1 (‘Very poor’) to 5 (‘Very good’). This measure has been successfully utilized to explore the relationship between transference, insight, and session quality outcomes. Internal consistency is not applicable for single‐item measures.

#### 2.5.2 Session Evaluation Scale (SES)

The SES is part of the Helping Skills Measure (Hill and Kellems 2002), and it gauges the perceived quality of therapy sessions, an essential aspect of session outcome. Originally, the SES featured four items scored on a five‐point Likert scale from 1 (‘Strongly disagree’) to 5 (‘Strongly agree’), with its validity indicated by correlations with session impact metrics (Hill and Kellems 2002). For this study, we used an enhanced five‐item version, which incorporates an item on overall session effectiveness (Lent et al. 2006). In this study, the SES demonstrated an internal consistency of McDonald's *ω* total of 0.78.

#### 2.5.3 Session Evaluation Questionnaire (SEQ)

The SEQ (Stiles 1980, 2002; Stiles et al. 1994) evaluates the impact of therapy and counseling sessions using 21 bipolar adjective scales in a 7‐point semantic differential format. Clinicians rate their feelings about the session or their mood post‐session, with higher scores reflecting a greater degree of the assessed construct. The SEQ evaluates four key dimensions: depth, smoothness, positivity, and arousal. Depth and smoothness relate to session evaluation, while positivity and arousal pertain to post‐session mood. Depth captures the perceived value and impact of the session; smoothness reflects the ease and comfort during the session. Positivity measures happiness, satisfaction, and confidence levels, while arousal gauges calmness or excitement. For our sample, the subscales' internal consistencies were McDonald's *ω* total of 0.87 for smoothness, 0.83 for depth, 0.65 for arousal, and 84 for positivity.

#### 2.5.4 Session Progress Scale (SPS)

The SPS (Kolden 1991), originally developed as a subscale of the Therapy Session Report (Orlinsky and Howard 1966), evaluate the impact of a specific session through four items rated on a Likert scale from 1 to 6, with lower scores reflecting greater progress. The specific descriptors for each quantitative continuous data vary by item, for example, “1” may denote “Completely helpful” and “6” “Not at all helpful.” The SPS has shown a test‐retest reliability of 0.75 and consistent validity by predicting treatment duration and outcomes (Kolden and Howard 1992). In this study, the SPS showed a McDonald's *ω* total of 0.85.

#### 2.5.5 Post‐Session Questionnaire (PSQ), Section B

The section B of the PSQ (Samstag et al. 1998) comprises four items evaluating alliance ruptures and their resolution within therapy sessions. The first item identifies any conflict, tension, disagreement, or misunderstanding occurred between patient and clinician in a certain session. If such an issue is reported, subsequent items measure the peak tension from 1 (‘Low’) to 5 (‘High’), the degree to which the issue was addressed by the end of that session from 1 (‘Not at all’) to 5 (‘Very much’), and the degree of resolution from 1 ('Not at all') to 5 (“Very much”). Lower tension and higher resolution correlate with better working alliance and session quality (Muran et al. 2009). Single‐item measures do not report internal consistency.

#### 2.5.6 Insight

The Insight scale (Gelso et al. 1991) is composed of three single items that evaluate, from the therapist's perspective, the level of intellectual, integrative, and overall insight reached by the patient during a therapy session. Insight encompasses the patient's accurate comprehension of the therapy material, including relationships, external functioning, and personal dynamics. Intellectual insight refers to understanding cause‐and‐effect relationships, while integrative insight links affect and intellect. Clinicians rate each item on a five‐point scale from 1 (‘None or slight’) to 5 (‘Very much’). Internal consistency is not reported for single‐item scales.

### 2.6 Sample Size

Determining the appropriate sample size for confirmatory factor analysis (CFA) depends on several factors, including the number of items, the strength of item‐factor relationships, model complexity, reliability, and the estimation methods employed (Kline and Little 2023; Streiner et al. 2015). For a scale validation study involving a noncomplex scale with reasonable indicator‐to‐factor ratios, such as the SPARQ‐C (two four‐item factors), a sample size of 150 participants is considered adequate to yield reliable and valid parameter estimates, ensuring a robust model fit (Brown 2015; Kline and Little 2023; Kyriazos 2018). When evaluating the correlations between the scale and other measures to establish various types of validity, similar sample size considerations are relevant. Typically, a sample size ranging from 100 to 200 is recommended to provide adequate statistical power for detecting meaningful correlations and confirming the scale's validity (Hair et al. 2022; Tabachnick et al. 2019). Therefore, for the current study, a sample size of 150 is deemed appropriate.

### 2.7 Statistical Analyses

The present analyses used a two‐step approach that checked basic assumptions and the number of major factors before shifting to confirmatory models using the structure found in the patient‐report version. The preliminary steps started with the Kaiser–Meyer–Olkin test and Bartlett's test of sphericity. A parallel analysis was conducted using various factor retention methods and was facilitated by the *EFAtools* R packages version 0.4.4 (Steiner and Grieder 2020). Then confirmatory factor analysis (CFA) was performed using a robust maximum likelihood estimator using the R package *lavaan* version 0.6–18 (Rosseel 2012). Multiple fit indices were estimated to provide a comprehensive evaluation of model fit (Hu and Bentler 1998; Kline and Little 2023). Specifically, the Comparative Fit Index (CFI) and Tucker–Lewis Index (TLI) compare the specified model to a baseline model, with values ≥ 0.95 generally indicating excellent fit. The Root Mean Square Error of Approximation (RMSEA) accounts for model parsimony, with values ≤ 0.06 suggesting close fit, though values ≤ 0.05 are sometimes preferred. The Standardized Root Mean Square Residual (SRMR) reflects the standardized difference between observed and predicted correlations, with values ≤ 0.08 considered acceptable, though values ≤ 0.06 indicate stronger fit. Internal consistency for each scale was measured using McDonald's *ω* total, Cronbach's *α*, and the average inter‐item correlation, with values calculated using the R package *psych* version 2.3.12 (Revelle 2024). The standard error of measurement (*SE*_*m*_) and the standard error of difference (*SE*_*d*_) were calculated to quantify the deviation in scores due to the measurement error. Criterion validity was assessed by correlating each SPARQ‐C scale with sociodemographic, clinical, and treatment variables, as well as validated measures of the therapeutic relationship and session outcomes, using Spearman's rank correlations to account for skewness and kurtosis, and adjusting *p*‐values via the Benjamini–Hochberg methods (Benjamini and Hochberg 1995). We evaluated the strength of correlations using the following thresholds: 0 to 0.19, very weak; 0.20 to 0.39, weak; 0.40 to 0.59, moderate; 0.60 to 0.79, strong; and 0.80 to 1, very strong (Stefana et al. 2025a), although these categories are somewhat arbitrary (Campbell 2021; Stefana et al. 2025a). The survey ensured complete data collection by requiring responses to all questions, resulting in no missing data.

### 2.8 Procedures

The Institutional Review Board of the University of North Carolina at Chapel Hill approved and exempted the procedures and materials used in this study (study number 23‐2656). Participants were recruited by email invitations sent to members of various professional associations and registries from February to May 2024. To qualify for participation, individuals needed to be licensed psychologists, counselors, psychotherapists, or analysts; and they were required to have at least one adult patient (18 years or older) currently under their professional care. Participants were instructed to consult their appointment records and select the most recent adult patient they had seen for an individual session. The survey, which focused on the last session with the selected patient, was conducted online through Qualtrics and took approximately 17 min to complete. Each participant was allowed to contribute only once.

## 3 Results

### 3.1 Preliminary Analyses

The Bartlett test of sphericity (*χ²*_[28]_ = 399, *p* < 0.001) and the Kaiser–Meyer–Olkin test (0.78) supported the suitability of the data for factor analysis.

### 3.2 Exploratory Factor Analysis

Parallel analyses with EFA, SMC, PCA, the Empirical Kaiser criterion, and Comparison Data all indicated two factors, so we conducted an EFA extracting these two factors. Items loaded adequately to strongly on their respective factors. The factors explained 52% of the variance, with positive affect accounting for 28% and negative affect 24%.

### 3.3 Confirmatory Factor Analysis

CFA results indicated that a single‐factor model provided an excellent fit for both scales. For the positive affect factor: χ^2^_[2]_ = 1.75, CFI = 1.00; TLI = 1.00, RMSEA = 0.00 (90% CI [0.00, 0.16]), and SRMR = 0.02. For the negative factor: χ^2^_[2]_ = 0.90, CFI = 1.00; TLI = 1.02, RMSEA = 0.00 (90% CI [0.00, 0.13]), and SRMR = 0.01. The two‐factor model that combined the two scales demonstrated an excellent fit for our data: χ^2^_[19]_ = 26.01, CFI = 0.98; TLI = 0.97, RMSEA = 0.05 (90% CI [0.00, 0.09]), and SRMR = 0.05.

### 3.4 Correlations Between Subscales

The positive affect and negative affect scales correlated *r* = 0.26 (*p* < 0.001).

### 3.5 Internal Consistency and Normal Distribution

The positive affect (*k* = 4) and negative affect (*k* = 4) scales demonstrated excellent internal consistency, with Cronbach's *α* of 0.83 and 0.77, McDonald's *ω* total of 0.84 and 0.77, and average inter‐item correlations of 0.55 and 0.44, respectively. For the entire SPARQ‐C (*k* = 8), internal consistency was reflected by McDonald's *ω* of 0.85, Cronbach's *α* of 0.79, and an average inter‐item correlation of 0.32.

The skewness and kurtosis values for the positive affect scale were within normal limits, recorded at −0.56 and −0.27, respectively. Conversely, the skewness and kurtosis for the negative affect scale were higher, at 1.93 and 4.78, respectively.

### 3.6 Score Precision

The mean scores for the positive and negative affect scales were 12.46 (SD = 2.69) and 1.83 (SD = 2.24), respectively. The percents of maximum possible (POMP) score were 78% and 11%, respectively. *SE*_*m*_ and *SE*_*d*_ were 1.08 and 1.53 for the positive affect scale and 1.07 and 1.52 for the negative affect scale.

### 3.7 Associations Between Scale Scores and Sociodemographic, Clinical, and Treatment Variables

As detailed in Table 3, both the positive and negative affect scale scores showed very weak correlations (*r* coefficients ranging from −0.13 to 0.21) with all patients' and clinicians' demographic data, patient features, and treatment characteristics listed in Tables 1 and 2.

**Table 3:** Criterion validity correlations with patient demographics, diagnoses, and objective therapy characteristics.

	Positive affect	Negative affect
Therapist age	−0.02	0.13
Therapist gender (Man higher)	−0.13	0.02
Therapist ethnicity	−0.00	−0.02
Professional background	−0.02	0.04
Psychotherapeutic approach	0.07	0.16
Years of post‐licensed experience	0.01	0.07
Weekly Time spent practicing therapy	0.00	0.03
Patient age	0.13	0.04
Patient gender	−0.08	−0.00
Patient ethnicity	0.08	0.01
Any psychiatric disorder	0.07	−0.00
Any anxiety disorder	0.18	−0.03
Any trauma‐ and stressor‐related disorders Any (unipolar) depressive disorder	0.19	−0.03
Any (unipolar) depressive disorder	−0.06	0.04
Any neurodevelopmental disorder	−0.00	0.00
Any personality disorder	0.00	0.00
Any eating disorder	0.14	0.06
Any bipolar or related disorder	−0.09	−0.04
Any other disorder	−0.09	−0.10
CGI	−0.15	0.14
GAF	0.21*	−0.13
Therapy length (months, ordinal; see prior table)	0.16	0.06
Session frequency (ordinal; see prior table)	0.05	0.14
Session attendance (in person, telephone, video)	−0.06	−0.06
Location (categorical; see prior table)	0.09	−0.01

*Note:* Coefficients are point‐biserial correlations for dichotomized variables, Spearman correlations for ordinal and continuous variables.

*p < 0.05, two‐tailed.

### 3.8 Criterion Validity

Table 4 details all the correlation coefficients computed for both the positive and the negative affect scales with the measures of therapeutic relationship and the measures of session outcome.

**Table 4:** Criterion validity correlations with validated scales.

	Scale score	Positive affect	Negative affect
**Therapeutic relationship measures**			
WAI‐SR‐T total score	67.22 (8.98)	0.64***	−0.36***
WAI‐SR‐T goal	20.85 (3.94)	0.51***	−0.28**
WAI‐SR‐T task	22.36 (3.24)	0.54***	−0.42***
WAI‐SR‐T bond	24.01 (3.02)	0.66***	−0.26**
RRI‐T total score	94.68 (10.77)	0.58***	−0.25**
RRI‐T genuineness	47.74 (5.89)	0.59***	−0.27**
RRI‐T realism	46.93 (5.51)	0.51***	−0.22**
CARE positive engagement	9.79 (1.65)	0.52***	−0.13
CARE enmeshed	2.25 (2.23)	0.23**	0.22*
CARE stuck	1.46 (1.69)	−0.23**	0.28**
ICB total amount	24.67 (3.84)	−0.13	0.29**
ICB positive	12.43 (2.69)	−0.06	0.20*
ICB negative	12.23 (1.82)	−0.15	0.28**
Tranference total amount	2.25 (1.13)	0.09	0.30***
Tranference positive	2.63 (1.27)	0.28**	0.13
Tranference negative	1.50 (0.86)	−0.02	0.31***
**Session outcome measures**			
Insight overall	3.85 (0.98)	0.34***	−0.11
Insight intellectual	3.90 (1.08)	0.25*	0.00
Insight integrative	3.66 (1.10)	0.39***	−0.16
SES	21.14 (3.09)	0.52***	−0.23*
Session quality	4.01 (0.84)	0.56***	−0.26**
SPS	14.09 (3.64)	−0.57***	0.23**
SEQ depth	3.06 (0.66)	0.47***	−0.24**
SEQ smoothness	4.19 (1.18)	0.37***	−0.29***
SEQ positivity	4.37 (0.90)	0.52***	−0.31***
SEQ arousal	2.84 (0.94)	−0.00	0.00
PSQ (yes)^a^	20 (13%)	−0.05	0.21
Degree of tension	2.40 (1.05)	−0.21	0.42
Extent issue addressed	3.60 (1.54)	0.26	0.11
Degree of resolution	3.15 (1.42)	0.12	0.01

^a^ Number of observations and percentage on the whole sample.

Coefficients are Spearman correlations

* *p* < 0.05

** *p* < 0.01

*** *p* < 0.001, two‐tailed.

#### 3.8.1 Therapeutic Relationship Measures

The SPARQ‐C positive affect scale was strongly and positively correlated with the bond dimension (*r* = 0.66) of the working alliance (WAI‐SR‐T) and with the genuineness dimension (*r* = 0.59) of the real relationship (RRI‐T). Furthermore, it was moderately correlated with the CARE positive engagement subscale (*r* = 0.52), the task (*r* = 0.54) and the goal (*r* = 0.51) dimensions of the alliance, and the realism dimension (*r* = 0.51) of the real relationship.

The SPARQ negative affect scale was weakly and positively correlated with the amount of negative transference (*r* = 0.31) and moderately and negatively correlated with the task dimension (*r* = −0.42) of the alliance.

#### 3.8.2 Session Outcome Measures

The SPARQ positive affect scale was moderately correlated with five measures of session outcome (i.e., Session quality, SES, SPS, SEQ depth, and SEQ positivity) (*r*s range = 0.47–0.57) and weakly‐to‐moderately correlated with Insight integrative (*r* = 0.39).

The SPARQ negative affect scale showed only very weak to weak correlations with measures of session outcomes. The highest correlation was *r* = −0.31 with the occurrence of any conflict or tension within the therapeutic dyad during the session (PSQ).

## 4 Discussion

The present article aimed to validate a clinician‐rated version of the SPARQ, previously only available in a patient self‐report version. The absence of a therapist version of this measure might be limiting, and we addressed this gap so that the patient's in‐session patterns of positive and negative affective reactions toward the therapist could begin to be measured dyadically from the perspective of both patient and clinician. Clinicians can integrate the SPARQ‐C into their regular practice as an assessment tool to identify their patients' subtle emotional shifts that might otherwise go unnoticed during the therapy, thereby facilitating timely interventions. Our findings support the use of the SPARQ‐C in both research and clinical settings, particularly for assessing the patient's subjective affective reactions towards their therapist and session‐level affective processes from the clinician's perspective.

The SPARQ‐C is identical to the original patient version of the SPARQ. Both versions consist of two four‐item factors. This condition in which one version perfectly mirrors the other one is ideal for getting a nice complement. Future research is required to assess how these measures interact and complement and to determine the distinct contributions of each. However, it is important to note that while the clinician version offers a convenient and practical means of assessment, it is based solely on the clinician's evaluation. Discrepancies between the clinician and patient versions may stem from factors such as countertransference (Stefana et al. 2020; Stefana et al. 2022; Stefana et al. 2022; Stefana & Youngstrom 2023), limitations in a therapist's empathy (Elliott et al. 2018), or difficulties in reading subtle emotional cues or understanding patients' lived experiences (Wehrens and Walters 2018), especially when patients are non‐disclosing, dissociative, or reluctant to share aspects of their emotional state. Conversely, therapists may sometimes perceive aspects of the patient's experience that the patient is unaware of or unwilling to report, leading to seemingly contradictory yet clinically complementary perspectives. In both cases, the presence of divergence between patient and clinician scores represents an important point for clinical reflection.

In the present study, the SPARQ‐C demonstrated strong psychometric properties. CFA fit values show that the SPARQ‐C possesses a robust factor structure, with items aligning well with the intended positive and negative affect dimensions. The excellent fit across multiple indices provides strong evidence for the construct validity of the scale. Internal consistency analysis confirmed the reliability of both the positive affect and negative affect scales. Content validity was supported by high factor loadings of each item onto the respective factor. Criterion validity was evidenced by significant correlations between the SPARQ‐C positive affect and both the WAI‐SR‐T and the RRI‐T, indicating that these constructs are theoretically and clinically related as expected. Similarly, the SPARQ‐C negative affect demonstrated a moderate correlation with the construct of negative transference. These findings are consistent with those from the SPARQ patient form. Notably, patients' feelings of security and comfort within the therapeutic relationship, as measured by the SPARQ‐C, were related to the therapist's positive experience of the relationship, as measured by the CARE positive engagement.

Regarding associations with session outcomes, the SPARQ‐C positive affect scale showed significant correlations with five different measures of session outcome. Conversely, the negative affect scales demonstrated only weak correlations with session outcomes.

Lastly, in line with previous research on the SPARQ, no significant differences emerged in the current study across demographic variables (both therapist and patient), clinical features of the patients (including psychiatric diagnosis), and psychotherapeutic treatment characteristics.

The SPARQ and SPARQ‐C are significant and distinctive contributions to psychotherapy research. They are psychometrically sound, short, and easy to administer in real‐world psychotherapeutic settings for both research and clinical purposes (Stefana et al. 2024 d). These measures are clinically sophisticated and specifically designed to assess the patient's subjective affective reactions towards their therapist and session‐level affective processes from both members of the therapeutic dyad. Their brevity makes them suitable for repeated measures assessments, reducing the measurement burden often encountered in complex psychotherapy studies.

The items directly focus on emotional states and reactions commonly experienced by the patient during a session, providing a measure of the quality of the therapeutic relationship. It is noteworthy that the original item pool of the SPARQ was composed of 131 items, which were reduced to the current 8 through sophisticated statistical analysis based on validation data collected from two different clinical samples of adult patients in individual therapy (*N*_sample A_ = 701 and *N*_sample B_ = 700). Therefore, the SPARQ and SPARQ‐C reflect the emotional configurations emerging in everyday psychotherapeutic practice. The questionnaire includes an equal number of positively and negatively valenced items, ensuring comprehensive coverage.

Despite decades of psychotherapy research focused on the relationship between the therapeutic relationship and session and treatment outcomes (Norcross 2011; Norcross and Lambert 2019), there is still considerable unexplained variance and key gaps in our understanding of the processes that lead to significant changes, both positive and negative. Dyadic studies of the various elements of the therapeutic relationship are necessary to more fully understand how these elements, both individually and in combination, unfold throughout psychotherapy and predict different trajectories and treatment outcomes. In this regard, adopting a dyadic perspective through the combined use of SPARQ and SPARQ‐C might enhance patient outcomes over time by offering a more comprehensive view of the therapeutic process. The utilization of the SPARQ and SPARQ‐C is expected to help fill this gap.

The present study has some limitations. First, the study had a cross‐sectional design. Responses might have shown different variability if a repeated measures design had been used, allowing for fluctuations in therapist perceptions. However, the SPARQ‐C was designed to assess the patient's reaction within a single session. This cross‐sectional nature limits our understanding of how affective dynamics evolve throughout treatment. Second, although the sample size was adequate for factor analyses and validity correlations, it was relatively small, and a larger sample would have been desirable. Additionally, the predominance of participants with psychodynamic/psychoanalytic orientations may limit the generalizability of these findings to other therapeutic modalities, such as cognitive‐behavioral or third‐wave approaches. Another limitation is the reliance solely on therapist‐report data (SPARQ‐C) without collecting complementary patient data (SPARQ). However, the primary goal of the study was to validate a clinician version of the questionnaire for use in future research. Future research should aim to collect and analyze data from both patient and clinician forms.

Despite these limitations, the validation of a clinician version of the SPARQ is overdue and will be useful for clinicians and researchers interested in the construct of affective reactions toward the therapist. Clinicians may find that regular administration of the SPARQ‐C aids in monitoring subtle shifts in patients' affective responses, potentially allowing for more responsive and adaptive treatment planning. Having a psychometrically strong clinician version to complement ongoing research will enrich the literature and inform future investigations. Further work is required to fully examine the SPARQ‐T. Supplementary reliability and validity data are needed to confirm the psychometric integrity of the questionnaire, and additional research will be necessary to assess its clinical and research utility. Longitudinal research is also required to investigate (a) how affective reactions evolve throughout therapy and predict different trajectories and outcomes, and (b) whether the SPARQ‐C can serve as an efficient tool for routine monitoring in psychotherapy. It will be particularly informative to explore these dynamics in diverse therapeutic settings (e.g., short‐term vs. long‐term therapy, psychoanalysis vs third‐wave cognitive behavioral therapy) as well as across different patient clinical and demographic features, thus paving the way for the implementation of more personalized and/or tailored interventions. The SPARQ and SPARQ‐C should be administered in tandem to provide a more comprehensive understanding of patients' in‐session emotional processes. This multi‐informant approach may reveal discrepancies or convergences between therapist and patient perceptions, thereby offering insights that could enhance treatment effectiveness over time. Given the importance of the therapeutic relationship in various settings beyond counseling and psychotherapy, both versions of the SPARQ should be validated in different clinical settings, such as psychiatric evaluation sessions and supportive nursing care sessions. Lastly, given the influence of cultural factors on emotional expression and therapeutic interactions, future work should focus on cross‐cultural adaptations and validations of the SPARQ‐C. Validating this instrument in international contexts will ensure its sensitivity to varying emotional and cultural dynamics, thereby broadening its applicability across different clinical populations.

## Conflicts of Interest

Eric A. Youngstrom has received royalties from the American Psychological Association and Guilford Press, and consulted about psychological assessment with Signant Health. He is the cofounder and Executive Director of Helping Give Away Psychological Science (HGAPS. org). Eduard Vieta has received grants and served as consultant, advisor or CME speaker for the following entities: AB‐Biotics, AbbVie, Adamed, Angelini, Biogen, Boehringer‐Ingelheim, Celon Pharma, Compass, Dainippon Sumitomo Pharma, Ethypharm, Ferrer, Gedeon Richter, GH Research, Glaxo‐Smith Kline, Janssen, Lundbeck, Medincell, Merck, Novartis, Orion Corporation, Organon, Otsuka, Roche, Rovi, Sage, Sanofi‐Aventis, Sunovion, Takeda, and Viatris, outside the submitted work. The other authors declare no conflicts of interest.

## Data Availability

The data that support the findings of this study are available from the corresponding author upon reasonable request.

## 1

*In‐Session Patient Affective Reactions Questionnaire–Clinician (SPARQ‐C) Form.*

The following is a series of statements that people in psychotherapy might use to describe how they feel toward their psychotherapist. Think about your last psychotherapy session, put yourself in your patient's shoes, and then read each statement and indicate how you think your patient felt during that session. Do not worry if responses appear to be inconsistent, as people often experience mixed and conflicting feelings.

**Table:**

Item nr.		Not at all	A little	Somewhat	A lot	Very much
1	They felt happy to see me.	0	1	2	3	4
2	They felt ashamed in my presence about their fantasy, desires, mindset, behavior, or symptoms.	0	1	2	3	4
3	They felt worried I couldn't help them.	0	1	2	3	4
4	They felt shy, like they wanted to hide from me or end the session early.	0	1	2	3	4
5	They felt afraid to spoke their mind, for fear of being judged, criticized, disliked by me.	0	1	2	3	4
6	They felt I cared about them.	0	1	2	3	4
7	They felt respected by me.	0	1	2	3	4
8	They felt appreciated by me.	0	1	2	3	4

Positive Affect items: 1, 6, 7, and 8. Negative Affect items: 2, 3, 4, and 5.
